# LogSpin: a simple, economical and fast method for RNA isolation from infected or healthy plants and other eukaryotic tissues

**DOI:** 10.1186/1756-0500-5-45

**Published:** 2012-01-19

**Authors:** Hila Yaffe, Kobi Buxdorf, Illil Shapira, Shachaf Ein-Gedi, Michal Moyal-Ben Zvi, Eyal Fridman, Menachem Moshelion, Maggie Levy

**Affiliations:** 1 Department of Plant Pathology and Microbiology, The Robert H. Smith Faculty of Agriculture, Food and Environment, The Hebrew University of Jerusalem, P.O. Box 12, Rehovot 76100, Israel; 2 The Robert H. Smith Institute of Plant Sciences, The Robert H. Smith Faculty of Agriculture, Food and Environment, The Hebrew University of Jerusalem, P.O. Box 12, Rehovot 76100, Israel

**Keywords:** RNA extraction, Infected tissue, Plant, Fungus, Aphids

## Abstract

**Background:**

Rapid RNA extraction is commonly performed with commercial kits, which are very expensive and can involve toxic reagents. Most of these kits can be used with healthy plant tissues, but do not produce consistently high-quality RNA from necrotic fungus-infected tissues or fungal mycelium.

**Findings:**

We report on the development of a rapid and relatively inexpensive method for total RNA extraction from plants and fungus-infected tissues, as well as from insects and fungi, based on guanidine hydrochloride buffer and common DNA extraction columns originally used for the extraction and purification of plasmids and cosmids.

**Conclusions:**

The proposed method can be used reproducibly for RNA isolation from a variety of plant species. It can also be used with infected plant tissue and fungal mycelia, which are typically recalcitrant to standard nucleic acid extraction procedures.

## Background

There are several known methods of RNA extraction. Most require reagents such as phenol-chloroform, which according to the US Environmental Protection Agency, are toxic and have a negative effect on the environment [1-3]. A rapid and less toxic option for RNA extraction is the use of commercial kits, but these are significantly more expensive than traditional phenol-chloroform extraction. The price of the extraction can become a limitation when there is a demand to extract RNA from a large number of samples.

Logemann et al. [4] described a common RNA extraction protocol which makes use of 8 M guanidine hydrochloride buffer to inhibit ribonucleases (RNases) [5], supplemented with 20 mM MES hydrate and 20 mM EDTA, and fresh addition of 0.0034% β-mercaptoethanol. In this protocol, proteins are removed by phenol/chloroform/isoamylalcohol (25:24:1). The RNA is precipitated by ethanol or isopropanol with 1 M acetic acid and washed with 3 M sodium acetate (pH 5.2) to dissolve low-molecular-weight RNA and contaminating polysaccharides, leaving the intact RNA as a pellet after centrifugation. The salts are removed by a final wash with 70% ethanol and the RNA pellet is subsequently dissolved in sterile water. This and similar protocols are inexpensive but are time-consuming and RNA yield varies among samples. In addition, the toxicity of the materials to human health and the environment must be considered.

The quality of RNA extracted from plant tissues using the above protocol is generally high. However, this is not the case with plant species that have high phenol or cellulose contents, from which RNA is difficult to extract. RNA is even harder to extract from fungus-infected tissues, showing only variable success, with very low relative yields and quality [6,7].

In this work, we report on a simple, economical, fast, and relatively non-toxic high-yielding method for RNA extraction, termed LogSpin. This method combines the RNA extraction protocol described by Logemann et al. [4] with a standard plasmid DNA extraction spin column. We eliminate the need for most toxic reagents and reduce the extraction time, making it possible to handle a large number of samples on the same day. The LogSpin method produces a high yield of clean, high-quality total RNA from tissues of a variety of plant species, including necrotic tissues and fungal mycelia, which are recalcitrant to RNA extraction by common protocols, as well as from insects. We show that the extracted RNA is suitable for cDNA synthesis and reverse transcriptase applications, including semi-quantitative and quantitative real-time RT-PCR.

## Results and discussion

### RNA extraction using plasmid DNA extraction columns

Spin columns contain a silica resin that selectively binds DNA/RNA, depending on the salt setting and other aspects influenced by the extraction method. Silica-based nucleic acid purification approaches make use of chaotrophic salts that denature proteins (including DNases and RNases) but also denature nucleic acids by disrupting their hydrogen bonding. This leads to selective binding of the nucleic acids to the silica resin in the column, and their effective separation from the rest of the sample. The nucleic acids are then washed with chaotropic salts to remove protein and pigment contaminants, and with ethanol to remove salts. After washing, nucleic acids are eluted from the column with water or low-salt solution, which induce its renaturation and thus eliminates their affinity for the silica resin.

Since all nucleic acid spin columns are essentially based on the same silica matrix technology, we set out to verify whether plasmid DNA columns can be used to purify RNA. The use of a plasmid DNA extraction column as an RNA-binding device for RNA extraction was first tested by comparing plasmid DNA extraction spin columns (QIAprep Spin Miniprep Kit, cat. no. 27106, Qiagen, Hilden, Germany) with RNA extraction spin columns from the commercial plant RNA extraction kit RNeasy (RNeasy Plant Mini Kit, Qiagen, cat. no. 74904). We compared the efficiencies of the two columns using reagents supplied with the RNeasy Plant Mini Kit, with some modifications. We extracted Arabidopsis plant tissue using 8 M guanidine hydrochloride buffer, a chaotropic salt that denatures proteins, thus inhibiting RNase, and denatures RNA so that under these conditions, the RNA will selectively bind to the silica resin in the column, separating it from the rest of the sample. The extraction buffer was also supplemented with 20 mM MES hydrate, to adjust the acidity for favored partitioning of RNA in the aqueous phase, and 20 mM EDTA, which also serves as an RNase inhibitor (β-mercaptoethanol was not added) [4]. In some cases, 96% ethanol (EtOH), which is known to precipitate nucleic acids [8], was also added 1:1 (v/v) to the samples prior to loading, to examine whether it improves RNA binding to the column. Homogenized samples (with and without 96% EtOH) were loaded onto the columns that were then treated with solutions from the RNeasy Plant Mini Kit. When the plant extracts contained ~48% EtOH, the plasmid DNA extraction columns were efficient as RNA-binding columns (Table 1). Without the addition of EtOH, no RNA was obtained at the end of the extraction process (Table 1), supporting the conclusion that EtOH is necessary for binding, i.e. in its absence the RNA molecules are washed away.

**Table 1:** RNA purification from Arabidopsis by plasmid DNA extraction column (pDNA) (QIAprep Spin Miniprep Kit, Qiagen) and RNA collection column (RNA) (RNeasy, Qiagen)

			RNA yield	Absorbance	
	Column	EtOH	μg/100 mg	260/280	260/230
1	pDNA	-	0.306	2.44	0.03

2	pDNA	+	12.46	2.06	2.14

3	RNA	-	0.138	1.59	0.68

4	RNA	+	11.48	2.08	2.09

**Figure 1:**
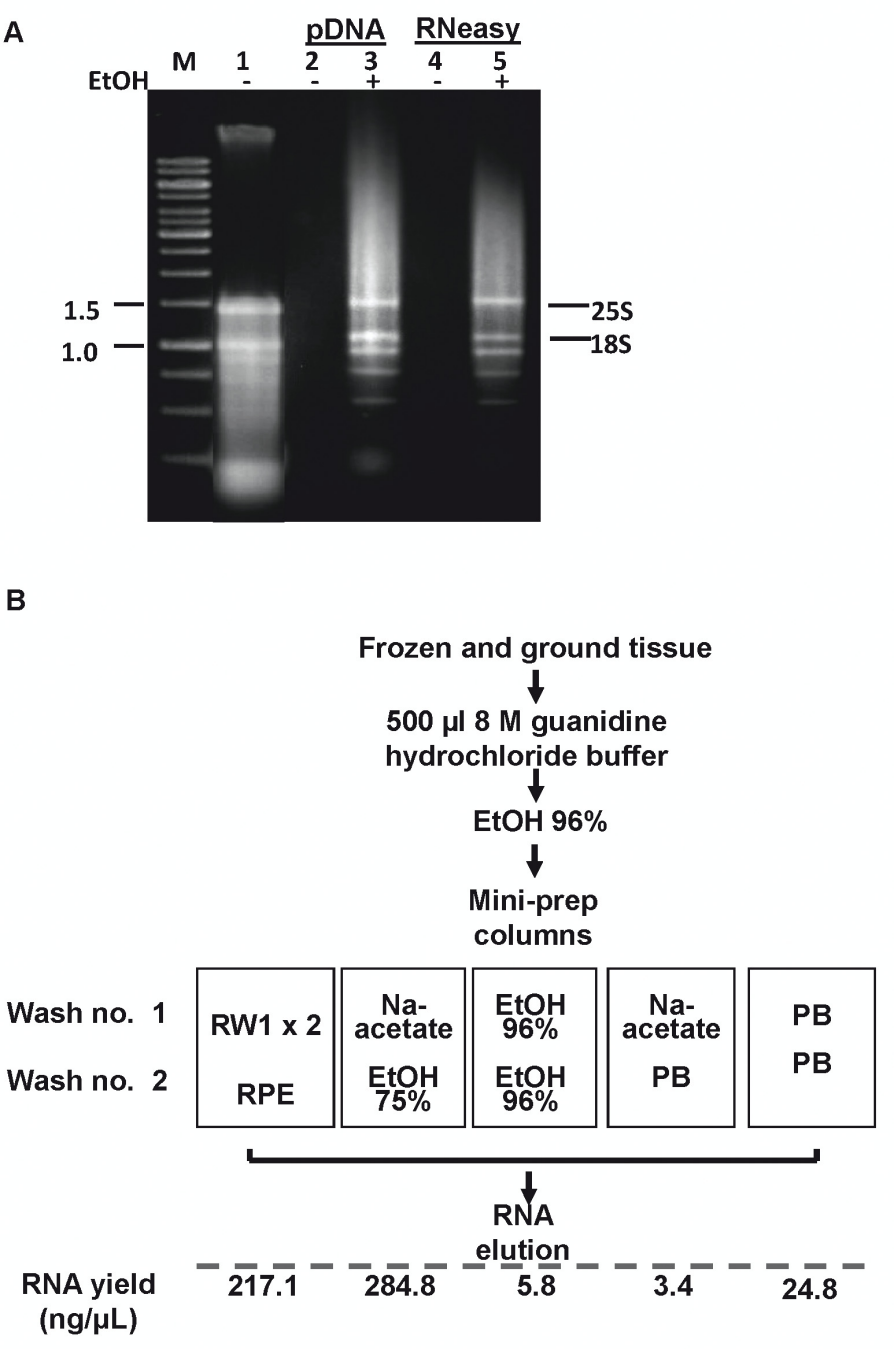
**Development of RNA extraction protocol**. **A**: RNA extracted from Arabidopsis leaves. Lane 1, RNA extraction using Logemann et al.'s protocol [4]. Lanes 2 and 3, RNA obtained by transfer through a plasmid DNA extraction column (QIAprep Spin Miniprep Kit). Lanes 4 and 5, RNA obtained from transfer through an RNA collection column (RNeasy). EtOH, 96% ethanol; M, DNA 1-kb ladder. **B**: RNA yield following extraction in 8 M guanidine hydrochloride buffer and 0.5 volume of 96% EtOH and transfer through a plasmid DNA extraction column (QIAprep Spin Miniprep Kit), followed by 2-3 washes in: (1) RW1 × 2 and RPE (RNeasy Plant Mini Kit), (2) 3 M Na-acetate and 70-75% EtOH; (3) two washes with 96% EtOH; (4) 3 M Na-acetate and PB (QIAprep); (5) two washes with PB. RNA yield was measured spectrophotometrically.

The purity of the extracted RNA was examined spectrophotometrically, by monitoring the absorbance ratios at OD_260_/OD_280 _and OD_260_/OD_230 _to estimate the amount of RNA (ng/μl) and levels of protein and salt in the samples, respectively. Equivalent results were obtained for RNA from the two column types (Table 1). We then examined the presence of ribosomal (r) RNA subunits by gel electrophoresis (Figure 1A), as an indicator of the intactness of the RNA product. Following our extraction procedure, we were able to detect the 25S and 18S ribosomal subunits as sharp bands on the gel (Figure 1A), supporting that there was little or no degradation of the extracted RNA.

**Figure 2:**
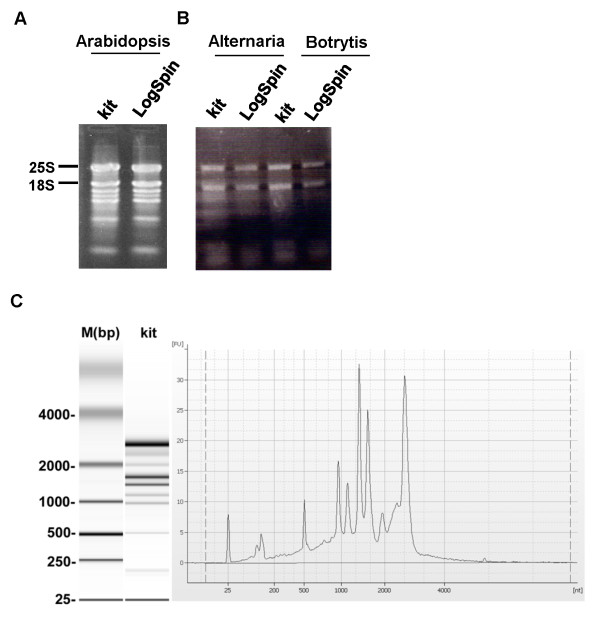
**RNA separation on 1% agarose gel**. **A**: RNA extracted from Arabidopsis leaves. Lane 1, RNA extracted using Qiagen RNeasy kit (kit) and lane 2, RNA extracted by LogSpin protocol. **B**: RNA extracted from *B. cinerea *(Botrytis) or *A. brassicicola *(Alternaria) mycelium. Lanes 1 and 3, RNA extracted using Norgen Plant/Fungi RNA Purification kit (kit). Lanes 2 and 4, RNA extracted using LogSpin protocol. **C**: Qualitative assessment of the integrity of a total RNA sample extracted using LogSpin protocol from tomato leaves by bioanalyzer. M (bp), DNA ladder in base pairs; kit, gel provided by the bioanalyzer kit.

Once we established that plasmid DNA spin columns can bind RNA with the use of commercial solutions (RNeasy Plant Mini Kit), we compared the efficiencies of five solution combinations for the washing step: (1) RW1 and RPE (RNeasy Plant Mini Kit buffers, Qiagen), (2) 3 M Na-acetate and 75% EtOH, (3) 96% EtOH, (4) 3 M Na-acetate and PB buffer (QIAprep binding buffer, Qiagen), and (5) PB only (Figure 1B). We found that the best non-commercial combination for RNA washing was 3 M Na-acetate followed by 75% EtOH: the chaotropic salt helped removing protein and colored contaminants, and ethanol removed the salts. None of the other tested combinations were suitable for our extraction method (Figure 1B). The total RNA obtained with the Na-acetate-EtOH wash was similar to that obtained using RNeasy washing solutions (Figure 1B), and was also shown to be intact, based on visualization of 25S and 18S ribosomal subunits and by assessment of total RNA integrity using an RNA 6000 nano chip (cat. no. 5067-1511, Agilent) run on an Agilent (Waldbronn, Germany) 2100 Bioanalyzer (Figures 1A and 2). All RNA species characteristic of leaf tissues- two peaks representing 25S and 18S cytoplasmic rRNA, three peaks corresponding to chloroplast rRNA and small RNA species peaks- could be detected on the Bioanalyzer electropherogram for tomato RNA extracted using our protocol (Figure 2) [9,10]. RNA integrity number (RIN) of our samples was 6.9 ± 0.2 [11].

### LogSpin extraction demonstrate plant RNA yields similar to those obtained with commercial kits

We compared our LogSpin protocol to those commonly used in our laboratory: the Tri-reagent protocol (Sigma-Aldrich, St. Louis, MO, USA), the RNeasy Plant Mini Kit for plant tissues and the Norgen kit for *Botrytis cinerea*. We extracted RNA from tomato seedlings and Arabidopsis leaves using the LogSpin, Tri-reagent and RNeasy protocols and obtained similar yields and purity (Table 2). RNA was extracted from petunia flowers using RNeasy, Tri-reagent and LogSpin: yields and purity levels were similar following the LogSpin protocol and the RNeasy kit. On the other hand, the LogSpin yield was lower than that obtained with the Tri-Reagent kit but the RNA purity was much higher in the former (Table 2), being clean enough to be applied for real-time PCR (unpublished data). These results indicate that RNA yield using the LogSpin protocol is comparable to that obtained using commercial kits or, when lower, is much purer.

**Table 2:** RNA purification from tomato and Arabidopsis leaves, petunia flowers and *Botrytis cinerea *mycelium by LogSpin protocol and by commercial kits

			RNA yield^1^	Absorbance	
	Organism	Protocol	μg/100 mg	260/280	260/230
1	Tomato	TRI^2^	31.3 ± 6.2	2.07 ± 0.01	2.27 ± 0.01

3	Tomato	RNeasy^3^	26.7 ± 13.3	2.11 ± 0.00	2.33 ± 0.04

2	Tomato	LogSpin	25.1 ± 1.8	2.14 ± 0.01	2.30 ± 0.01

4	Arabidopsis	TRI^1^	21.1 ± 5.9	2.13 ± 0.00	2.05 ± 0.12

5	Arabidopsis	RNeasy^3^	16.3 ± 3.8	2.10 ± 0.01	2.28 ± 0.01

6	Arabidopsis	LogSpin	15.5 ± 3.6	2.15 ± 0.01	2.29 ± 0.01

7	Petunia	TRI^2^	37.8 ± 5.5	1.90 ± 0.06	1.30 ± 0.25

8	Petunia	RNeasy^3^	14.7 ± 1.0	2.07 ± 0.02	2.02 ± 0.14

9	Petunia	LogSpin	13.6 ± 0.5	2.06 ± 0.02	2.21 ± 0.01

10	*B. cinerea*	KIT^4^	31.0 ± 3.4	2.12 ± 0.00	2.14 ± 0.00

11	*B. cinerea*	LogSpin	83.3 ± 18.9	1.87 ± 0.07	2.05 ± 0.08

^1^RNA yield ± SE (n = 3-10).

^2^Tri-reagent, Sigma-Aldrich.

^3^RNeasy, Qiagen.

^4^Norgen.

### LogSpin extraction leads to higher RNA yield from infected plant tissue and fungi than extraction by commercial kits

RNA extraction from *B. cinerea*-infected and uninfected Arabidopsis leaves using the commercial RNeasy Plant Mini Kit yielded significantly lower RNA yield from infected tissue (9.3 *± *1.7 μg) than from its uninfected counterpart (16.3 *± *3.8 μg; *t*-test, *P *< 0.05)(Table 3). In comparison, using the LogSpin protocol, we obtained twice the RNA yield (15.8 *± *4.2 μg) from Arabidopsis leaves infected with *B. cinerea*, similar to the amount obtained from uninfected tissue (Table 3). Furthermore, we were able to obtain clean RNA from fungal mycelia (*B. cinerea *and *Alternaria brassicicola*) using the LogSpin protocol (Figure 2A, B): RNA yield from *B. cinerea *mycelium using this protocol was higher, albeit not significantly, than that produced by the Norgen commercial fungal RNA-extraction kit (Norgen Biotek Corporation, Canada) (Table 2). We hypothesize that our proposed method produces clean, high-quality RNA from necrotic tissues because guanidine hydrochloride can dissolve the tissue and utilization of the column prevents accumulation of tissue aggregates in the sample.

**Table 3:** RNA purification by LogSpin, Tri-reagent (TRI) and Qiagen RNeasy kit (KIT) from uninfected and infected Arabidopsis tissue

		RNA yield^1 ^μg/100 mg	Absorbance	
			
	Extracted		260/280	260/230
1	Uninfected - LogSpin	15.5 ± 3.6	2.15 ± 0.01	2.29 ± 0.01

2	Infected - LogSpin	15.8 ± 4.2	2.16 ± 0.01	2.30 ± 0.02

3	Uninfected - KIT	16.3 ± 3.8*	2.10 ± 0.01	2.28 ± 0.01

4	Infected - KIT	9.3 ± 1.7*	2.13 ± 0.01	2.22 ± 0.05

5	Uninfected - TRI	21.1 ± 3.4^&^	2.13 ± 0.00	2.05 ± 0.07

6	Infected - TRI	10.3 ± 0.81^&^	2.12 ± 0.04	2.02 ± 0.05

^1^RNA yield ± SE, (n = 3-6).

*Statistically significant, *t*-test, *P *= 0.007.

^&^Statistically significant, *t*-test, *P *= 0.036.

### The LogSpin method can be used for a diverse range of eukaryotic organisms, including fungus-infected plant tissue

We assessed our LogSpin protocol with tissues from a variety of organisms: leaves of Arabidopsis, tomato and rose, barley meristems and petunia flowers, as well as the aphid *Bemisia tabaci *and the fungal mycelia of *B. cinerea *and *A. brassicicola*. In all of these experiments, high yields of clean total RNA were produced (Table 4). We also succeeded in obtaining routinely higher yields of clean RNA from fungus-infected leaf tissues, which are known to be recalcitrant to common RNA-extraction protocols (Table 3). An exception was with rose leaf samples: these contain high levels of phenols and polysaccharides which make the RNA extraction very complicated and time-consuming [12,13]. Using the LogSpin protocol, we obtained very low RNA yields but the extraction was much easier and the obtained RNA was clean enough for real-time PCR analyses (see Additional file 1). Since the LogSpin protocol is based on guanidinium salt, it is not recommended for use with tissues containing high phenolic and secondary metabolites if high RNA yields are needed. since the salts are ineffective at dissociating RNA from non-protein complexes and might interfere with the RNA resuspension and clog the columns [14,15]. In such cases, a guanidinium-free protocol is preferred [16,17].

**Table 4:** RNA purification by LogSpin from a variety of organisms

			Absorbance	
			
	Organism	RNA yield^1 ^μg/100 mg	260/280	260/230
1	Tomato^2^	25.1 ± 1.8	2.14 ± 0.01	2.30 ± 0.01

2	Barley^2^	12.3 ± 1.2	2.06 ± 0.04	2.30 ± 0.04

3	Arabidopsis^2^	15.5 ± 3.6	2.13 ± 0.02	2.22 ± 0.05

4	Petunia^3^	13.6 ± 0.5	2.06 ± 0.02	2.21 ± 0.01

5	Rose^2^	1.78 ± 0.2	2.46 ± 0.18	2.07 ± 0.03

6	*B. cinerea*^4^	83.3 ± 19	1.87 ± 0.07	2.05 ± 0.08

7	*A.brassicicola*^5^	64.8 ± 15	2.14 ± 0.06	2.29 ± 0.11

8	*B. tabaci*^6^	32.5 ± 2.3	1.94 ± 0.06	1.67 ± 0.10

^1^RNA yield ± SE, (n = 3-10).

^2^leaves; ^3 ^flowers; ^4^mycelium; ^5^mycelium and spores.

^6 ^μg ± SE from 100 aphids, n = 3.

Our protocol can also be used with RNA-extraction protocols that require phenol for membrane lysis, such as RNA extraction from nuclei. The plasmid DNA column can be loaded with the liquid phase obtained from the phenol extraction. In fact, the DNA spin columns can be coupled with any front-end RNA or nucleic acid extraction method that uses ethanol precipitation to purify RNA.

### Reverse transcriptase applications

**Figure 3:**
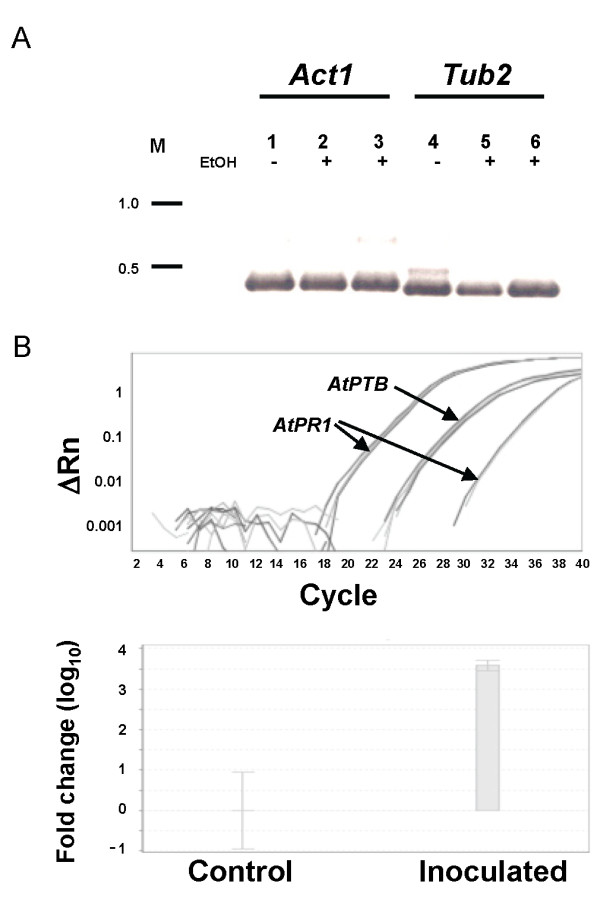
**RT-PCR and quantitative RT-PCR analysis**. **A**: PCR analysis of cDNA transcribed from RNA extracted by the different columns. RT-PCR, Lanes 1 and 4, cDNA template was transcribed from RNA extracted using the common RNA extraction protocol [4] without column extraction step. Lanes 2 and 5, cDNA template was transcribed from RNA produced using the plasmid DNA extraction column. Lanes 3 and 6, cDNA template was transcribed from RNA produced using the RNA collection column (RNeasy Plant Mini Kit). EtOH, 96% ethanol added to samples before transfer through columns. *Actin 1 *(*Act1*, lanes 1-3) and *β-Tubulin 2 *(*Tub2*, lanes 4-6) primers were used. M, DNA 1-kb ladder. **B**: Quantitative real-time RT-PCR on cDNA transcribed from RNA produced using LogSpin protocol, amplification plot (upper panel) and fold change of *AtPR1 *gene normalized to *AtPTB1F *on samples from inoculated versus uninoculated (control) Arabidopsis leaves. Relative *AtPR1 *gene expression was calculated by the 2^-ΔΔCt ^method (ΔRn).

To verify the suitability of the RNA extracted from Arabidopsis leaves by our method for reverse transcriptase applications, 1000 ng RNA was treated with DNase I followed by reverse transcriptase reaction (see Methods). RNA stability after DNase I treatment was analyzed by gel electrophoresis: the RNA was found to be highly stable at 37°C for 30 min and at 72°C for 15 min. cDNA products obtained from the reverse transcriptase reaction were used in a PCR with primers for *A. thaliana Actin 1 *(*Act1*) and *β-tubulin 2 (Tub2*) genes (Additional file 2: Table S1), which can distinguish between genomic DNA and cDNA (see Methods), and showed high expression levels of the cDNA product, indicating that the RNA contained intact mRNA molecules and no DNA contamination (Figure 3).

We compared RNA extraction using our protocol on different plasmid mini prep columns (Qiagen QIAprep Spin Miniprep Kit and the HiYield Plasmid Mini Kit from RBC Bioscience, Taipei, Taiwan). From the results, we assume that the columns have the selective ability (low cutoff) to bind RNA nucleic acids preferentially over genomic DNA. Plasmid DNA extraction columns are designed to bind small DNA nucleic acid fragments (plasmids up to 10,000 bp). Finally, real-time PCR on cDNA synthesized from DNase I-treated Arabidopsis and rose RNA extracted by our LogSpin protocol showed consistently high-quality results (Figure 3B, C and Additional File 1). This indicates that the total RNA extracted with the LogSpin protocol is highly pure, remains intact and can be applied for downstream applications.

### Benefits of RNA extraction based on plasmid DNA extraction columns

The first advantage of the LogSpin protocol is economical: according to the prices provided by companies that are marketing RNA purification kits in Israel, the range is 3.75 to 12.2 USD per sample. The price of extracting one sample using the LogSpin protocol was 0.98 USD, ca. ~25% of the cost of the least expensive commercial kit. This price includes the solutions, the extraction columns and the eppendorf tubes.

The second advantage is the relative efficiency of this protocol, which makes it suitable for RNA extraction from a large number of samples (12-18) in less than 1 h (after plant tissue grinding). This allows several rounds of RNA production in the same day, providing numerous samples for treatment or use in further applications (eg., DNase I treatment, RT-PCR, for example) at the same time.

The third advantage of this protocol is the absence of phenol/chloroform/isoamylalcohol mixture and β-mercaptoethanol, compounds that are harmful to both humans and the environment (see Additional file 3).

Thus, not only is our RNA extraction method useful for plant tissues, including infected plant tissues, insects and fungal mycelia, which are normally recalcitrant to RNA extraction, it is also economical, simple and quick, and reduces exposure to harmful chemicals in the laboratory, to the benefit of workers' health and the environment.

## Conclusions

In this work, we report a simple, economical, fast, and relatively non-toxic high-yielding method for RNA extraction. We demonstrate the use of our method for RNA extraction from a variety of plant, fungal and insect tissues, and its reproducibility for RNA isolation from infected plant tissues, which are difficult to extract by other methods. The LogSpin method produces a high yield of clean, high-quality total RNA that is suitable for cDNA synthesis and reverse transcriptase applications, including semi-quantitative and quantitative real-time RT-PCR.

## Methods

### Plant material and growth conditions

*Arabidopsis thaliana *ecotype Ws-0 seeds were sown in soil (30% peat, 30% vermiculite, 20% tuff and 20% perlite) and vernalized at 4°C for 2 days before transferring to a controlled environment. Plants were kept in the growth room at 18°C under an 8/16 h light/dark photoperiod using fluorescent lamps (Osram L 36 W/840, Lumilux Cool White, Munich, Germany). Tomato, petunia and rose plants were grown in the greenhouse at 25°C under natural daylight. Barley plants were collected from the field.

### Fungal strains, growth and inoculation method

*B. cinerea *strain B05.10 and *A. brassicicola *isolated in Israel were grown on potato dextrose agar (PDA, Difco, France) in a controlled-environment chamber at 22°C under illumination with fluorescent and incandescent light, at a photofluency rate of approximately 120 μmol/m^2 ^s and 12 h day length. Conidia were harvested in sterile distilled water and filtered through four layers of sterile gauze to remove hyphae. For inoculation, conidial suspensions were adjusted to 3000 conidia/μl. *B. cinerea *conidial suspensions were prepared in half-strength filtered (0.45-μm) grape juice. Infected leaves were collected 72 h post-inoculation for RNA extraction.

### Chemicals for RNA extraction

Guanidine hydrochloride for molecular biology (≥ 99% pure), MES hydrate, minimum 99.5% titration, and diethyl pyrocarbonate (DEPC) were from Sigma-Aldrich. EDTA was supplied from J.T. Baker (Deventer, The Netherlands), and Na-acetate was purchased from Merck (Serono, Germany). All chemicals were dissolved in DEPC-treated water.

### Grinding the plant tissue

Tissue was inserted into a safety-locked eppendorf tube with two glass beads (diameter: 5-10 mm), frozen immediately in liquid nitrogen, ground in Tissue Lyser (Qiagen, CA, USA) to a fine powder and then kept at -80°C for further use. Until addition of the first solution, tissue samples were kept frozen. Afterward, samples were kept on ice.

### Common RNA extraction protocol

The extraction protocol was as described by Logemann et al. [4] with some modifications. Phenol/chloroform/isoamylalcohol (25:24:1) was prepared in a falcon tube 1 day before RNA extraction, sealed, covered with aluminum foil (the solution is sensitive to light) and left overnight in a fume closet. On the day of the experiment, 0.17 μl β-mercaptoethanol was added to 500 μl guanidine hydrochloride buffer (8 M guanidine hydrochloride, 20 mM MES hydrate and 20 mM EDTA made in the fume hood and sterilized), which was then added to 70 to 100 mg ground Arabidopsis leaves in a safety-locked eppendorf tube and mixed well. The supernatant was moved to a new microcentrifuge tube (liquid phase without the glass beads) filled with 400 μl of the lower (liquid) phase only of phenol/chloroform/isoamylalcohol. The suspension was mixed well for ~5 min by vortexing and centrifuged at 4°C, at maximum speed, for 10 min. The upper (aqueous) phase was collected in a new microcentrifuge tube and the same volume of cold isopropanol was added. The suspension was quickly vortexed and centrifuged at 4°C, at maximum speed, for 10 min. The liquid phase was carefully extracted and appropriately discarded, and the pellet was washed with 500 μl 3 M Na-acetate pH 5.2 (a short vortexing helped resuspend the pellet) and centrifuged at 4°C, at maximum speed for 5 min. The pellet was then washed with 70% EtOH (short vortexing to resuspend the pellet) and centrifuged at 4°C, at maximum speed for 5 min. At the end of the washing step, the pellet was dried at room temperature in a laminar flow hood, resuspended in 30 μl DEPC-treated water at 60°C, and left in a 60°C incubator for 10 min. RNA (1000 ng) was treated with DNase I and reverse-transcribed to produce cDNA. RNA yield and purity, before and after DNase I treatment, was measured spectrophotometrically (NanoDrop ND-1000 spectrophotometer, NanoDrop Technologies, Wilmington, DE, USA).

### RNA extraction by plasmid DNA extraction column

Guanidine hydrochloride buffer (500 μl; 8 M guanidine hydrochloride, 20 mM MES hydrate and 20 mM EDTA) was added to 70 to 100 mg frozen and ground Arabidopsis leaf tissue in a safety-locked eppendorf tube and vortexed for 5 to 15 s. The liquid phase (without glass beads) was transferred to a new microcentrifuge tube and centrifuged at 4°C, at maximum speed, for 20 min to sediment the ground leaf tissue as described above. The clear supernatant was then transferred to a new microcentrifuge tube containing 1:1 v/v (~250 μl) 96% EtOH, quickly vortexed and then loaded onto the plasmid DNA extraction column (QIAprep Spin Miniprep Kit, Qiagen, Hilden, Germany or HiYield Plasmid Mini Kit, RBC Bioscience). The plasmid DNA extraction column was assembled in a microcentrifuge tube, and centrifuged at 8,000 *g *for 45 s. The liquid flow-through was appropriately discarded. The column was washed twice: first with 450 μl 3 M Na-acetate, to remove polysaccharides, proteins and pigments, and then with 320 μl 70% EtOH to remove salts. Between and after washes, the column was centrifuged at 8,000 *g *for 45 s and the liquid flow-through removed. The column was dried by centrifugation at maximum speed for 2 min. For elution of the RNA from the column, 30 to 40 μl of DEPC-treated water at 60°C were added directly to the column membrane, incubated for 2 min at room temperature and centrifuged at 8,000 *g *for 2 min. To increase RNA yield, this step was repeated with the liquid flow-through. RNA yield and purity, before and after DNase I treatment, were measured spectrophotometrically and by gel electrophoresis.

### RNA measurements and quality control

RNA yield and purity were measured by absorbance at OD_260_, OD_260/280 _and OD_260/230 _and analyzed by gel electrophoresis at 100 V in a 1% agarose gel in 1× TAE buffer (40 mM Tris acetate, 1 mM EDTA). Gels were stained by incubation in ethidium bromide for 20 min, followed by washing in TAE buffer for 20 min, and exposure to UV light. RNA integrity was determined with an RNA 6000 nano chip run on an Agilent 2100 Bioanalyzer, with ~250 ng RNA loaded on the chip.

### cDNA synthesis and RT-PCR

RNA (1000 ng) was treated with DNase I (New England Biolabs, Ipswich, MA, USA) and 1000 ng of DNA-free RNA was reverse-transcribed to cDNA (EZ-First Strand cDNA Synthesis Kit for RT-PCR, Biological Industries, Beit Haemek, Israel). The products of the reverse transcription (cDNA) were detected by PCR analysis with *Act1 *and *Tub2 *primers (Additional file 2: Table S1), which on a cDNA template generate 431-bp and 411-bp products, respectively, while on a DNA template, product fragments are 670 bp and 1000 bp long, respectively. PCR cycles were: denaturation step at 94°C for 2 min, followed by 35 cycles of denaturation at 94°C for 30 s, annealing at 60°C for 30 s and elongation at 72°C for 60 s. A final extension step was performed at 72°C for 10 min. cDNA tubes were kept at -20°C and RNA tubes were kept at -80°C.

### Quantitative real-time RT-PCR (qRT-PCR) analysis

qRT-PCR was performed with the SYBR master mix and StepOne real-time PCR machine (Applied Biosystems, Foster City, CA). The thermal cycling program was as follows: 95°C for 20 s; 40 cycles of 95°C for 3 s and 60°C for 30 s. Relative fold change of *AtPR1 *gene normalized to *AtPTB1F *on samples from infected versus uninfected Arabidopsis leaves was calculated by the 2^-ΔΔCt ^method. The primer sequences are listed in Additional file 4: Table S2.

### Statistical analysis

T-tests were performed only when data was normally distributed and the sample variances were equal. Otherwise Mann-Whitney Rank Sum Test was performed. Significance was accepted at *P < 0.05 *and is noted in the text or table captions. All experiments shown here are representative of at least three independent experiments with the same pattern of results.

## Abbreviations

LogSpin: Logemann protocol coupled with spin columns; RT-PCR: Reverse transcription polymerase chain reaction; qRT-PCR: Quantitative real-time RT-PCR; EtOH: Ethanol; rRNA: Ribosomal RNA; PDA: Potato dextrose agar; DEPC: Diethyl pyrocarbonate.

## Competing interests

The authors declare that they have no competing interests.

## Authors' contributions

LM, MM and LF designed the experiments. HY, KB IS, EGS and MMBZ performed the experiments. ML and KB wrote the manuscript. All authors read and approved the final manuscript.

## Supplementary Material

Additional file 1 **Quantitation Report**. Click here for file

Additional file 2 **Table S1**. Primers used in RT-PCR. Click here for file

Additional file 3 **Toxicity affects of phenol/chloroform/isoamylalcohol and of the β-mercaptoethanol**. Click here for file

Additional file 4 **Table S2**. Primers used in qRT-PCR. Click here for file
