# Supplementary material for: LogSpin: a simple, economical and fast method for RNA isolation from infected or healthy plants and other eukaryotic tissues
Source: BMC Res Notes. 2012 Jan 19;5:45. doi: 10.1186/1756-0500-5-45 (PMC3282632; doi:10.1186/1756-0500-5-45)
Supplement: Additional file 1 — Quantitation Report. [file 1756-0500-5-45-S1.PDF]

Additional File 1

Quantitation Report

Experiment Information

Run Name	260510 ACTIN LEAVES
Run Start	26/05/2010 11:30:30
Run Finish	26/05/2010 13:11:10
Operator	Ben
Notes	
Run On Software Version	Rotor-Gene 1.7.75
Run Signature	The Run Signature is valid.
Gain Green	9.67

Quantitation Information

Threshold	0.0192
Left Threshold	10.000
Standard Curve Imported	No
Standard Curve (1)	conc= 10^(-0.324*CT + 11.581)
Standard Curve (2)	CT = -3.082*log(conc) + 35.695
Reaction efficiency (*)	1.11073 (* = 10^(-1/m) - 1)
M	-3.08231
B	35.6949
R Value	0.99464
R^2 Value	0.9893
Start normalising from cycle	1
Noise Slope Correction	Yes
No Template Control Threshold	0%
Reaction Efficiency Threshold	Disabled
Normalisation Method	Dynamic Tube Normalisation
Digital Filter	Light
Sample Page	actin
Imported Analysis Settings	

Profile

Cycle	Cycle Point
Hold @ 95°c, 15 min 0 secs	
Cycling (45 repeats)	Step 1 @ 94°c, hold 10 secs
	Step 2 @ 60°c, hold 30 secs
	Step 3 @ 72°c, hold 20 secs, acquiring to Cycling A([Green][1][1])

Melt (72-95°c) , hold secs on the 1st step, hold 5 secs on next steps, Melt A([Green][1][1])	

Raw Data For Cycling A.Green

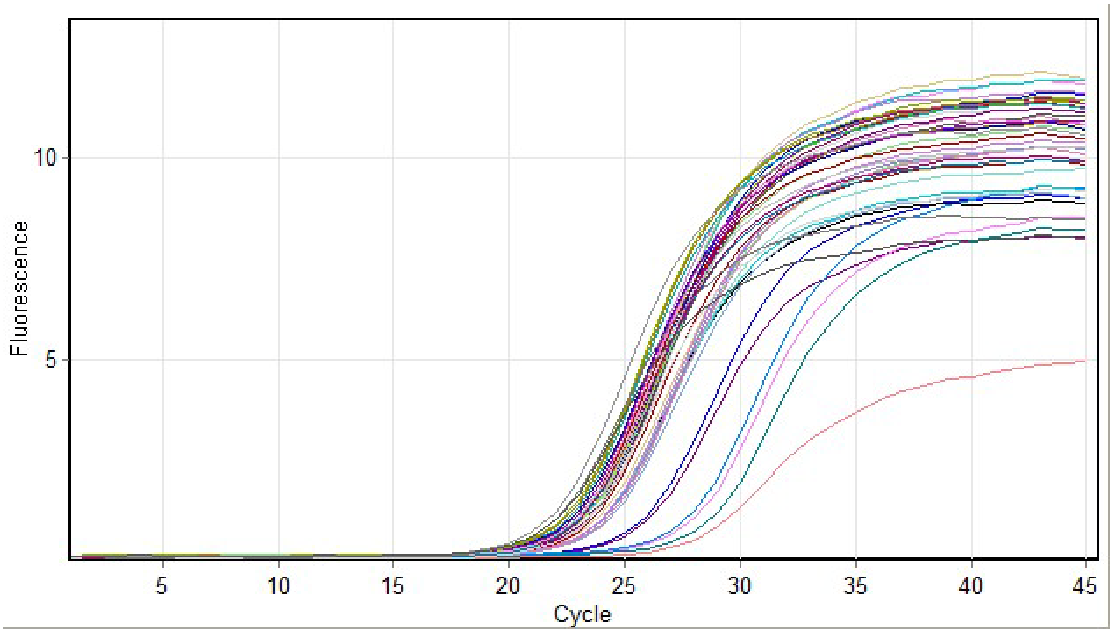

Quantitation data for Cycling A.Green

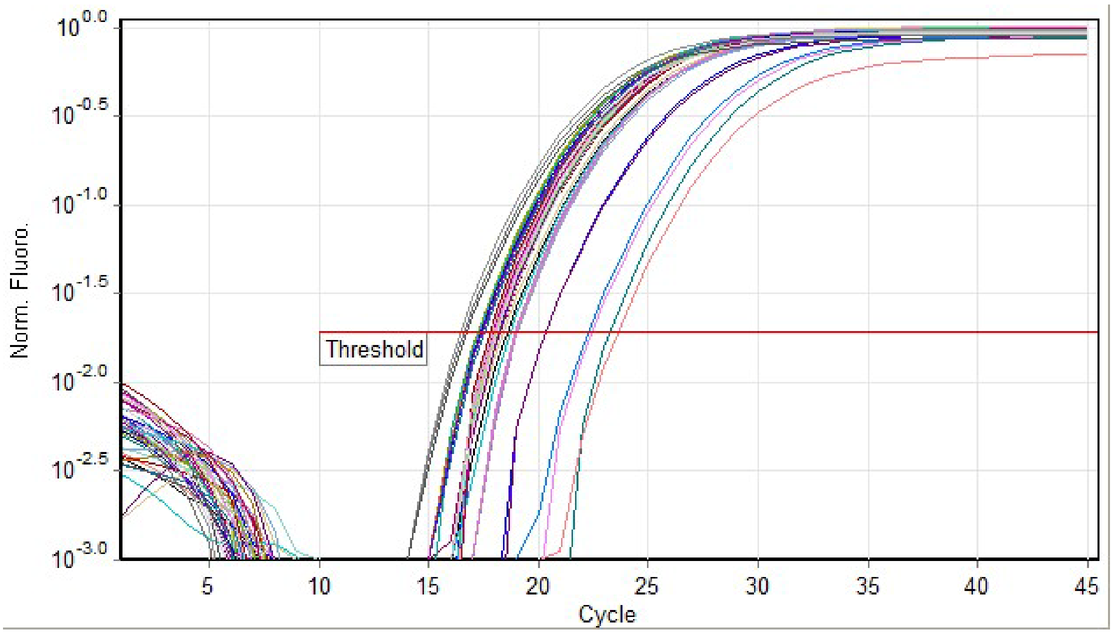

Standard Curve

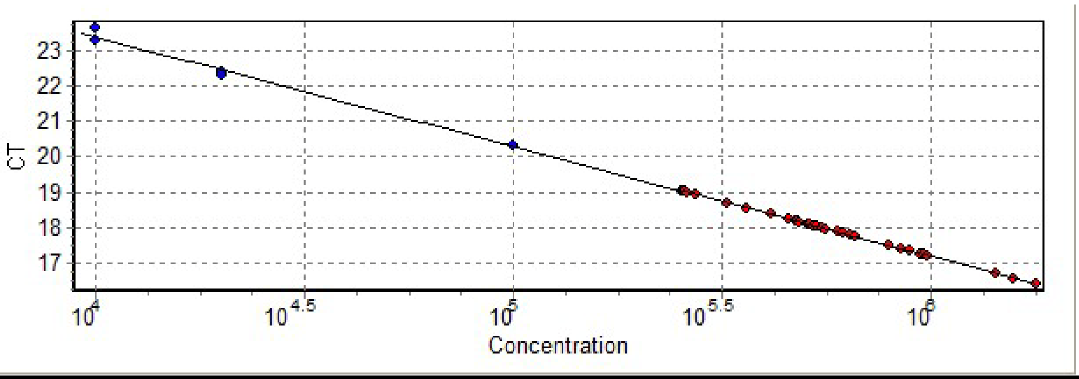

No.	Colour	Name	Type	Ct	Given Conc (copies/ul)	Calc Conc (cop
3		x100	Standard	20.32	100,000	97,523
4		x100	Standard	20.31	100,000	98,030
5		x500	Standard	22.41	20,000	20,462
6		x500	Standard	22.27	20,000	22,698
7		x1000	Standard	23.25	10,000	10,881
8		x1000	Standard	23.62	10,000	8,279
11		wt1	Unknown	18.56		362,948
12		wt1	Unknown	18.70		325,624
13		wt1	Unknown	18.37		416,838
14		wt2	Unknown	19.00		260,428
15		wt2	Unknown	19.03		255,386
16		wt2	Unknown	19.04		253,154
17		wt3	Unknown	19.02		256,100
18		wt3	Unknown	18.93		273,854
19		wt3	Unknown	19.01		259,184
20		gus1	Unknown	18.25		456,533
21		gus1	Unknown	18.06		525,575
22		gus1	Unknown	18.00		552,009
26		gus3	Unknown	18.17		485,655
27		gus3	Unknown	18.17		485,849
28		gus3	Unknown	18.25		455,441
32		p6-2	Unknown	18.18		482,180
35		p6-3	Unknown	18.10		509,268

No.	Colour	Name	Type	Ct	Given Conc (copies/ul)	Calc Conc (cop
36		p6-3	Unknown	18.20		474,357
37		p6-3	Unknown	17.97		562,458
44		p11-3	Unknown	18.06		525,365
45		p11-3	Unknown	18.04		533,178
46		p11-3	Unknown	18.10		511,508
50		p12-2	Unknown	17.80		640,326
51		p12-2	Unknown	17.84		620,211
52		p12-2	Unknown	17.75		663,206
53		p12-3	Unknown	17.22		987,175
54		p12-3	Unknown	17.25		963,825
55		p12-3	Unknown	17.28		942,154
56		p13-1	Unknown	17.51		791,732
57		p13-1	Unknown	17.36		891,329
58		p13-1	Unknown	17.41		852,378
59		p13-2	Unknown	17.89		598,815
60		p13-2	Unknown	18.09		515,812
61		p13-2	Unknown	17.84		620,459
62		p13-3	Unknown	16.42		1,792,362
63		p13-3	Unknown	16.59		1,577,542
64		p13-3	Unknown	16.72		1,428,930
3-8, 11-22, 26-28, 32, 35-37, 44-46, 50-64		aCTIN	Group			

NEG (NTC) - Sample cancelled due to NTC Threshold.
NEG (R. Eff) - Sample cancelled as efficiency less than reaction efficiency threshold.
