# Supplementary material for: LogSpin: a simple, economical and fast method for RNA isolation from infected or healthy plants and other eukaryotic tissues
Source: BMC Res Notes. 2012 Jan 19;5:45. doi: 10.1186/1756-0500-5-45 (PMC3282632; doi:10.1186/1756-0500-5-45)
Supplement: Additional file 2 — Table S1. Primers used in RT-PCR. [file 1756-0500-5-45-S2.PDF]

Additional File 2: Table S1 Primers used in the RT-PCR

Gene	Genbank accession no.	Primer sequence (5'→3')	Fragment length (bp)	
			cDNA	DNA
Actin1 (Act1)	AT2G37620	F: TGGAACTGGAATGGTTAAGGCTGG<br>R: TCTCCAGAGTCGAGCACAATACCG	434	670
β-Tubulin 2 (Tub2)	AT5G62690	F: TTCTCGATGTTGTTCGTAAGGAAGC<br>R: AGCTTTCGGAGGTCAGAGTTGAGTT	411	1000
