# Supplementary material for: LogSpin: a simple, economical and fast method for RNA isolation from infected or healthy plants and other eukaryotic tissues
Source: BMC Res Notes. 2012 Jan 19;5:45. doi: 10.1186/1756-0500-5-45 (PMC3282632; doi:10.1186/1756-0500-5-45)
Supplement: Additional file 3 — Toxicity affects of phenol/chloroform/isoamylalcohol and of the β-mercaptoethanol. [file 1756-0500-5-45-S3.PDF]

Additional File 3

Toxicity affects of phenol/chloroform/isoamylalcohol and of the β-mercaptoethanol

The third advantage of this protocol is the absence of the toxic phenol/chloroform/isoamylalcohol mixture and of the β-mercaptoethanol. Phenol (C_6HSOH) is produced commercially and has many uses which bring it into direct contact with humans. Inhalation by breathing contaminated air and dermal exposure through skin contact is highly irritating to the skin, eyes and mucous membranes and can induce eczema, inflammation, discoloration, necrosis, sloughing and gangrene. Inhalation alone adversely affects the lungs, causing hyperemia, infarcts, bronchopneumonia, purulent bronchitis, and hyperplasia of the bronchial tissues. Long-term inhalation exposure to phenol in animal studies has shown effects on the liver and kidneys, and on the respiratory, cardiovascular and central nervous systems (http://www.lakes-environmental.com/toxic/PHENOL.HTML). The US Occupational Safety and Health Administration (OSHA) (Babich & Davis, 1981) The American Conference of Governmental Industrial Hygienists (ACGIH) and The National Institute for Occupational Safety and Health (NIOSH) all recommend limiting exposure to phenol. Chloroform ($CHCl_3$) is a volatile member of the trihalomethane group (Aggazzotti *et al.*, 1995) which is widely used as an organic solvent (Kasai *et al.*, 2002; Yamamoto *et al.*, 2002). It is ubiquitously present in the air, drinking water, and some foodstuffs (Kasai *et al.*, 2002; Yamamoto *et al.*, 2002; Nagano *et al.*, 2006) and enters the body by inhalation, oral and dermal routes of exposure (Aggazzotti *et al.*, 1995; Kasai *et al.*, 2002; Yamamoto *et al.*, 2002; Nagano *et al.*, 2006). Chloroform is also classified as a possible human group 2B carcinogen by the International Agency for Research on Cancer (IARC) and the Japan Society for

Occupational Health (JSOH), and as a confirmed animal carcinogen with unknown relevance to humans by the ACGIH (Yamamoto *et al.*, 2002). The ACGIH and the JSOH recommend an occupational exposure limit (Kasai *et al.*, 2002). According to the Carcinogenic Potency Database (CPDB) at Berkeley University, chloroform has been found carcinogenic to rats and mice (http://potency.berkeley.edu/chempages/CHLOROFORM.html). Chloroform is also a major environmental contaminant, formed in the chlorination of community drinking water, in the cooling of water from power plants and in the process of bleaching paper, and is thus a potential hazard to the health of workers and community residents (Kasai *et al.*, 2002). Isoamylalcohol ($C_5H_{11}OH$) is a colorless flammable liquid that reacts violently with strong oxidants and reducing agents and can react with hydrogen trisulfide, creating an explosion hazard. The substance can be absorbed into the body by inhalation and by ingestion. It is irritating to the eyes, the skin and the respiratory tract, and if ingested, may affect the central nervous system. β-Mercaptoethanol ($HOCH_2CH_2SH$), also known as 2-hydroxy-1-ethanethiol or thioglycol, is a colorless liquid with a characteristic odor. The substance reacts with oxidants and metals and upon heating, it decomposes to toxic sulfur oxide gases. Like all of the aforementioned substances, it can be absorbed into the body by inhalation of its vapor, through the skin and by ingestion. Short-term exposure irritates the eyes, skin, and respiratory tract, and it may have detrimental effects on the central nervous system.

References

Aggazzotti G, Fantuzzi G, Righi E and Predieri G. 1995. Environmental and Biological Monitoring of Chloroform in Indoor Swimming Pools. *Journal of Chromatography A* **710**(1): 181-190.

Babich H and Davis DL. 1981. Phenol: A Review of Environmental and Health Risks. *Regulatory Toxicology and Pharmacology* **1**(1): 90-109.

Kasai T, Nishizawa T, Arito H, Nagano K, Yamamoto S, Matsushima T and Kawamoto T. 2002. Acute and Subchronic Inhalation Toxicity of Chloroform in Rats and Mice. *Journal of Occupational Health* **44**(4): 193-202.

Nagano K, Kano H ,Arito H, Yamamoto S and Matsushima T. 2006. Enhancement of Renal Carcinogenicity by Combined Inhalation and Oral Exposures to Chloroform in Male Rats. *Journal of Toxicology and Environmental Health, Part A: Current Issues* **69**(20): 1827 - 1842.

Yamamoto S, Kasai T, Matsumoto M, Nishizawa T, Arito H, Nagano K and Matsushima T 2002. Carcinogenicity and Chronic Toxicity in Rats and Mice Exposed to Chloroform by Inhilation. *Journal of Occupational Health*. 283-293.
