# Supplementary material for: LogSpin: a simple, economical and fast method for RNA isolation from infected or healthy plants and other eukaryotic tissues
Source: BMC Res Notes. 2012 Jan 19;5:45. doi: 10.1186/1756-0500-5-45 (PMC3282632; doi:10.1186/1756-0500-5-45)
Supplement: Additional file 4 — Table S2. Primers used in qRT-PCR. [file 1756-0500-5-45-S4.PDF]

Additional File 4: Table S2 Primers used in the qRT-PCR

Gene	Genbank accession no.	Primer sequence (5'→3')	Fragment length (bp)
AtPTB1F	AT3G01150	F:GATCTGAATGTTAAGGCTTTTAGCG<br>R:GGCTTAGATCAGGAAGTGTATAGTCTTG	60
AtPR1	AT2G14610	F: GCCTTACGGGGAAAACTTA<br>R: CTTTGGCACATCCGAGTCT	60

At, Arabidopsis thaliana.
